# Technical and technological constraints facing Tanzania leather value chain: a snapshot of intervention measures

**DOI:** 10.1186/s42825-022-00095-2

**Published:** 2022-08-10

**Authors:** Cecilia Rolence China, Rahel Elibariki, Jamal Msami, Stephen Mwombela, Lugano Wilson

**Affiliations:** 1 Tanzania Industrial Research and Development Organization, P. O. Box 23235, Dar es Salaam, Tanzania; 2 REPOA, 157 Mgombani Street Regent Estate, P. O. Box 33223, Dar es Salaam, Tanzania

**Keywords:** Leather value chain, Hides and skins, Constraints, Interventions

## Abstract

The leather value chain starts with livestock, the key source of hides and skins, and the main raw materials for the industry. Despite having the second-largest livestock population in Africa, Tanzania is only the 11th largest exporter of leather on the continent, underlining the disproportionate contribution of the sector to the economy. This study presents the results of a recent assessment aimed at identifying performance bottlenecks in Tanzania's leather sector. The assessment took place in October–December 2020 and involved 10 tanneries, 30 leather goods factories and 11 service providers/institutions. Findings reveal that 90% of tanneries are dissatisfied with the quality of hides and skins due to brand marks, flay cuts, skin diseases, and inadequate curing. These supply chain bottlenecks are further compounded by skill deficiencies among processors, poor waste management, and limited awareness about laws, policies and regulations governing trade in the sector. At the downstream end of the value chain, the trade in leather and related articles is facing competition from high importation of low-grade products like plastic shoes and second-hand products. This paper recommends that efforts to increase leather's economic and trade value need to focus on upgrading Tanzania’s production, processing, branding and marketing capacities.

**Graphical Abstract:**

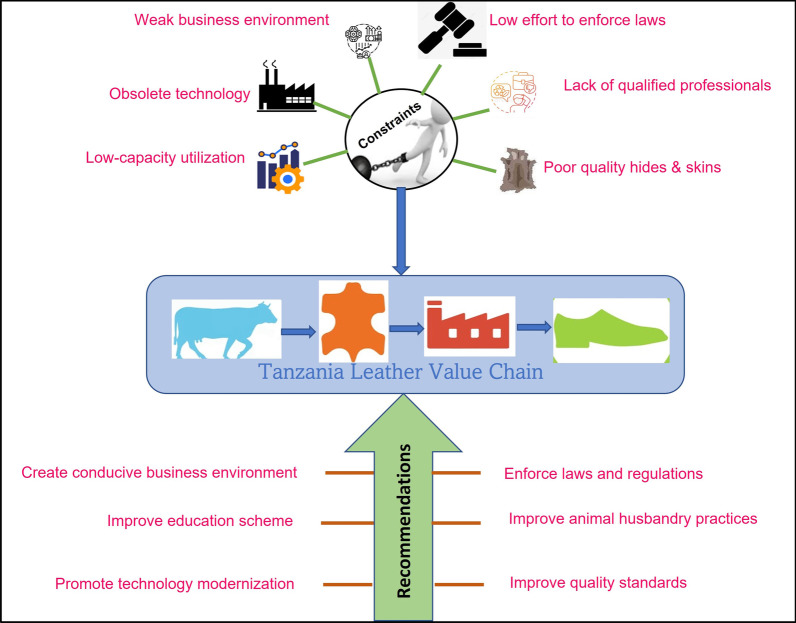

## Introduction

The global leather value chain is a complex system [1]. It starts with the recovery of hides and skins from slaughtered animals, followed by the conversion of the hides and skins into leather in tanneries [2]. This process usually requires substantial investment in equipment and the use of more than 160 kinds of chemicals [3]. The leather processing stage is then followed by the manufacture of leather products, which in developing countries is carried out in small labor-intensive and medium scale enterprises (SMEs) with less need for substantial investment in equipment and there are larger capital-intensive factories [4]. The resultant leather product then goes to the end-user, where it may be recycled to other valuable products at the end of its use [5, 6]. Therefore, the marketing of intermediate and end products at different stages in the value chain is the key to success in the modern leather products business [7].

The leather value chain is a peculiar agro-processing sector because it depends on another value chain, which is animal production. Its main input relies on animal production rates and the ability to collect and preserve the pelts. Principally, the leather industry is built on meat production worldwide [8]. Therefore, it relieves the food industry of what would be a major problem of disposing of waste hides and skins [9]. According to the National Bureau of Statistics, Tanzania is home to 32.2 million cattle, 18.8 million goats and 5.3 million sheep [13] that produced about 7,425,131 hides and skins in 2020. Connecting such livestock wealth with the fast-growing global population indicates the huge potential of the leather sector in raising the national economy. However, the sector supplies 90% hides and skins and only 10% of semi-finished leather to the global market [14]. At the same time, there is a high demand for leather products globally, as indicated in the market forecast report produced by Expert Market Research (EMR), that the global footwear market value reached almost USD 235.5 billion in 2020, further expected to grow to nearly USD 281.2 billion by 2026.

The current Government's political will to revamp the leather sector is a key asset to grow the sector [10]. So far, the Government has demonstrated various efforts, such as facilitating the establishment of two new tanneries (Kilimanjaro International Leather Industry Company Limited and Ace Leather Tanzania Limited). The sector also enjoys the conducive investment infrastructure, including an attractive business environment such as access to special economic zones and improved water and electricity infrastructure [11, 12].

Despite all the potentials available, the leather value chain is still underperforming in Tanzania. Compared to other agricultural export products such as coffee, cashewnut and cloves, the export contribution of the leather sector is almost negligible. According to the data from the National Bureau of Statistics [13], the export volume and export value of hides, skins and leather is 0.55% and 0.27% of the total agricultural commodity exported in 2019. The data reveals that 96% of the export volume is from raw hides and skin, indicating a low level of value addition. Thus, to contribute to unlocking barriers facing the sector, the Tanzania Industrial Research and Development Organization (TIRDO) surveyed the leather sector in 2020. The aim was to identify constraints that hinder the leather sector's performance and, after that, propose solutions. This paper, therefore, presents major findings of the Tanzania leather sector study.

## Methodology

### Sample size and sampling technique

The survey sample covered encompassed regions with the most leather industries to ensure accurate representation. The sample was selected by bias method (purposive sampling) based on the number, level and type of leather industries in that region. Consequently, the survey was carried out in the regions of Dar es Salaam, Morogoro, Arusha, Dodoma, Kilimanjaro, Coast and Mwanza. Stakeholders for focus group discussion were selected based on their strong influence on the leather sector. They include the Ministry of Livestock and Fisheries Development (MLFD), the Ministry of Industry and Trade (MIT), Tanzania Bureau of Standards (TBS), Leather Association of Tanzania (LAT), Tanzania Trade Development Authority (TanTrade), Tanzania Revenue Authority (TRA) and National Environment Management Council (NEMC).

### Data collection

The study employed a research methodological approach to collect both primary and secondary data. For primary data collection, the employed methods were quantitative and qualitative. The quantitative approach deployed questionnaires administered to leather industry owners, while the qualitative approach applied was a focus group discussion with leather industry stakeholders. Secondary data collection involved collecting the total list of factories and SMEs in the leather sector from trusted literature and the list of chemicals used in the leather sector from relevant Tanzanian institutions.

### Data analysis

The Statistical Package for Social Sciences (SPSS) and excel Programme analyzed the data gathered from the survey. The views and opinions of interviews and focus group discussions were analyzed as presented in the findings and discussion section.

## Results and discussion

Tanzania leather sector is one of the main sectors to impact the national industrialization agenda [12]. However, many challenges still face the sector, especially the quality of hides and skins and processed leather. Despite the Government and other stakeholders' commitment and concern with the sector, it is anticipated that some intervention measures are still needed to ensure that the sector is beneficial to the country [14]. On these bases, this study compiles useful information and recommendations of the hides and skins value chain from leather processors and leather product makers that could be useful for improving the sector.

### Current status of Tanzania leather value chain

#### Leather industries scales in Tanzania

Tanneries and leather product industries of Tanzania are categorized, as shown in Table 1. This categorization is based on the SMEs policy 2003 [15], primary data collected from the visited establishments during fieldwork, secondary information collected from the Ministry of Livestock and Fisheries, Ministry of Industry and the Trade and Tanzania Leather Product Producers Association (TALEPPA). The findings revealed that small and medium scale enterprises dominate the leather industry of Tanzania. Specifically, the leather product industries are highly dominated by SMEs. As mentioned by the stakeholders, this is due to the shortage of financial means to afford modern machines for expanding their factories. Therefore, the lack of capital is one of the hurdles preventing SMEs engaged in leather and leather goods manufacturing from graduating to large industries.

**Table 1:** Category and type of leather industries in Tanzania. Source: Ministry of Livestock and Fisheries, Ministry of Industry and Trade and Tanzania Leather Product Producers Association (TALEPPA), field survey data

Category	Leather tanneries	Leather goods makers
Large	4	5
SMEs	6	159

#### Nature of ownership

**Fig. 1:**
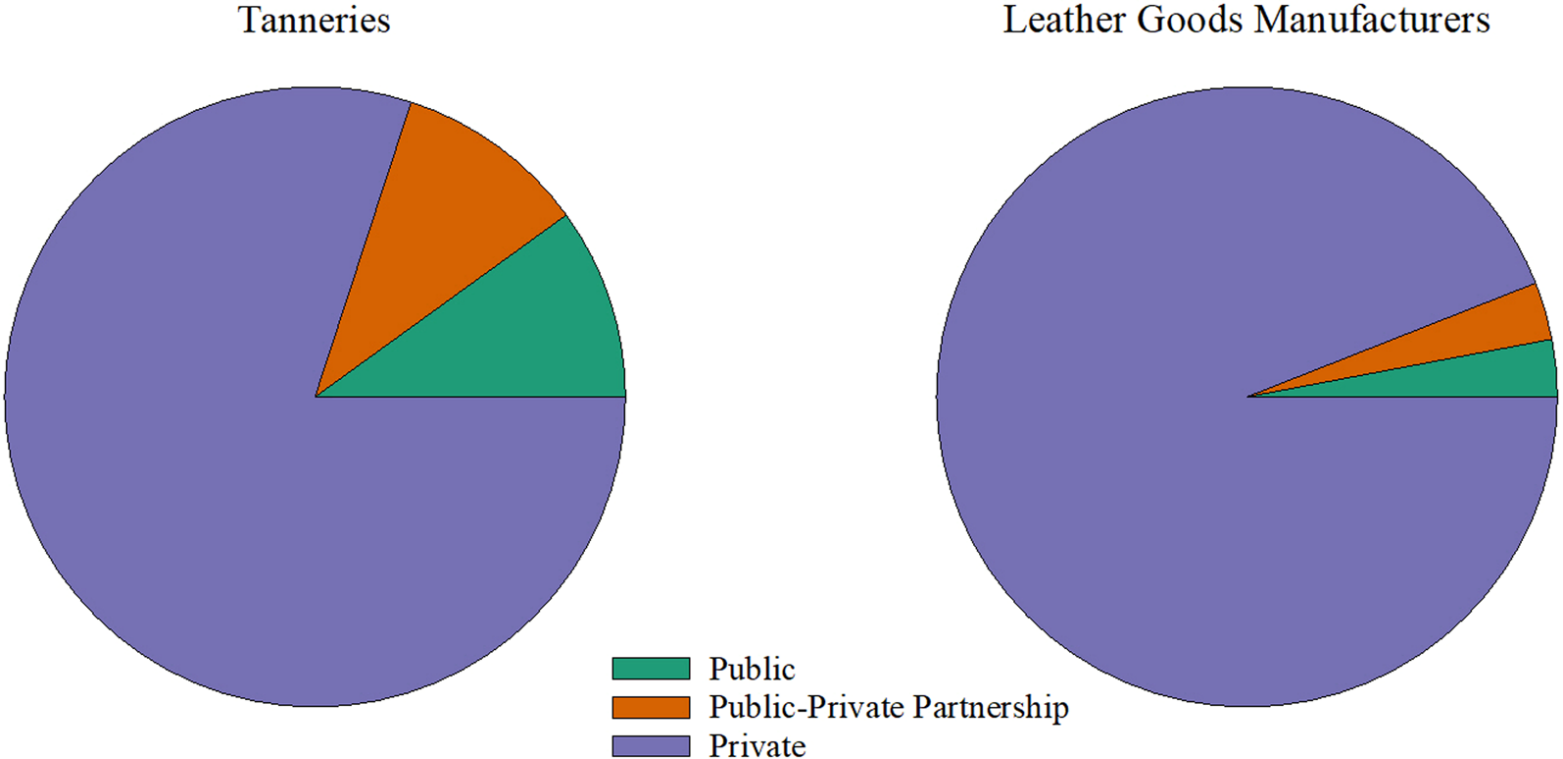
Ownership of leather industries by either public, private or partnership

**Fig. 2:**
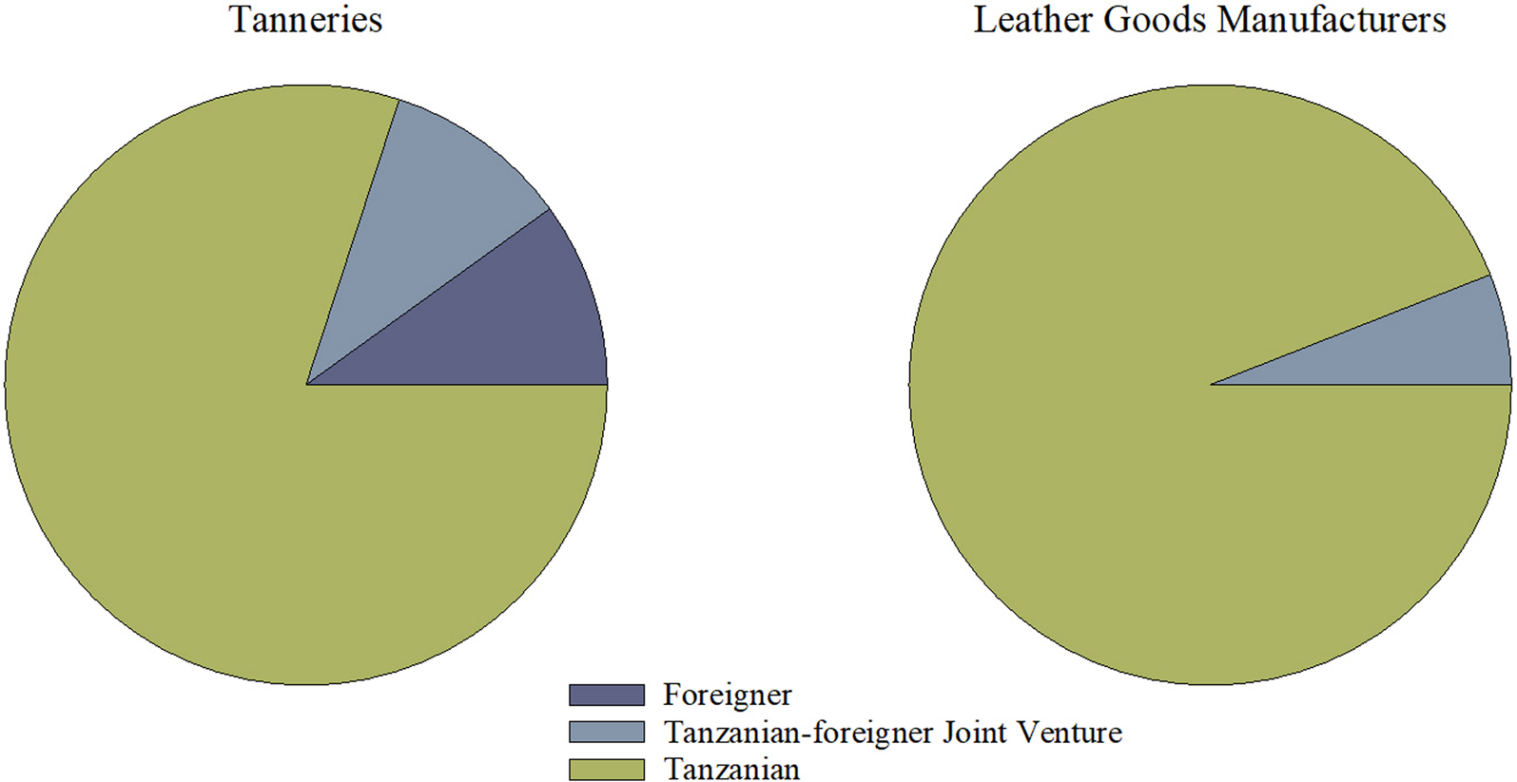
Ownership of leather industries by origin

Most of the established leather and leather goods industries are privately owned by Tanzanians, while the public and Private–Public Partnership (PPP) own a minority, as shown in Figs. 1 and 2. The history of leather industry ownership and the policies since Tanzania gained independence might have influenced the observed nature of ownership. Though the import substitution industrialization (ISI) policy (1961–1967) promoted private sector ownership of industries with little government participation to encourage industrial growth, the adoption of the Arusha declaration in 1967 demanded state ownership of major means of the product [16, 17]. Since the leather industries were developed during this time, all of them were state-owned, till 1980 when privatization took place [18]. Since then, the private sector has dominated the leather sector because the subsequent policies, particularly Sustainable Industrial Development Policy for Tanzania (SIDP 2020) and Integrated Industrial Development Strategy (IIDS) 2025 adopted in 1996 and 2010, respectively, actively promoted private ownership of the industries [17, 19]. Apart from that, National Investment Act 1997 that was enacted to promote and protect private investment by providing tax holidays, subsidies and low taxes on corporate profits, might have contributed to the observed situation [17].

Ownership by a PPP in Tanzania is governed by the National Private Public Partnership policy of 2009, Public–Private Partnership Act No.18 of 2010 and Public–Private Partnership Regulations of 2011 to spearhead and nurture/lead PPP to its fullest for benefiting the economy. According to Nwokorie [20], PPP ownership of investments offers a better way to finance infrastructure without adding burden to the public debt. Farquharson [21] added that PPP provides many benefits, including access to finance, access to technology, people and skills, transfer of risks, and business development. Tanzania's leather sector is missing these opportunities by having fewer industries owned under the partnership between private and public entities. This was observed during the survey that only few of the establishments are PPP owned, while majority are privately (Fig. 1).

In the category of ownership by origin, foreigners were observed to own the few establishments as compared to Tanzanians (Fig. 2). Although Tanzania is one of the most preferred destinations for foreign investment in Africa [22], low levels of industrial development, environmental concerns, lack of transparency and poor compliance with legislation are some of the identified barriers to investment for foreigners. Other obstacles are enacting laws without private sector consultation, ineffective regulations and stringent labour regulations to support a dynamic labour market [23, 24]. These barriers to Foreign Direct Investments (FDI) have existed for almost a decade now. The same was reported by United Nations Conference on Trade and Development (UNCTAD) [22] that ranked Tanzania below average in Africa on FDI attractiveness due to infrastructure and openness variables and certain government factors (principally payroll taxes, tax evasion and irregular payments).

#### Raw material supply and quality

The leather value chain in Tanzania starts at farms where livestock are the key source of hides and skins, which are the raw materials for the leather industry. In the slaughterhouses, flayers recover hides and skins from a slaughtered animal. The supplied hides and skins are of low quality due to poor animal husbandry practices. The presence of non-mechanized slaughterhouses and unskilled flayers also contributes to the low quality of hides and skins.

The findings show that about 95% of animals kept in Tanzania are indigenous breeds, leading to thin and small size hides and skins. Ngowi et al. [25] reported similar findings. Extreme defects characterize the hides and skins produced in Tanzania. About 90% of leather processors are not satisfied with the quality of hides and skins supplied to their tanneries. They acknowledged that the materials are damaged by brand marks, flay cuts, skin diseases and inadequate curing (rotting). The small size and thinness also affect the quality of hides and skins. These findings match those reported by Mbassa et al. [26] that 84% of animals in Tanzania are branded, which downgrade hides and skins quality. The same study highlighted the prevalence of flay cuts in the produced leather due to lack of equipment and trained personnel. Thus, as per the Guardian newspaper reporter [27], the hides and skins produced in Tanzania are known to be of low grades (III-V).

### Constraints in the Tanzanian leather value chain

#### Underutilization of tanneries' installed capacity

**Fig. 3:**
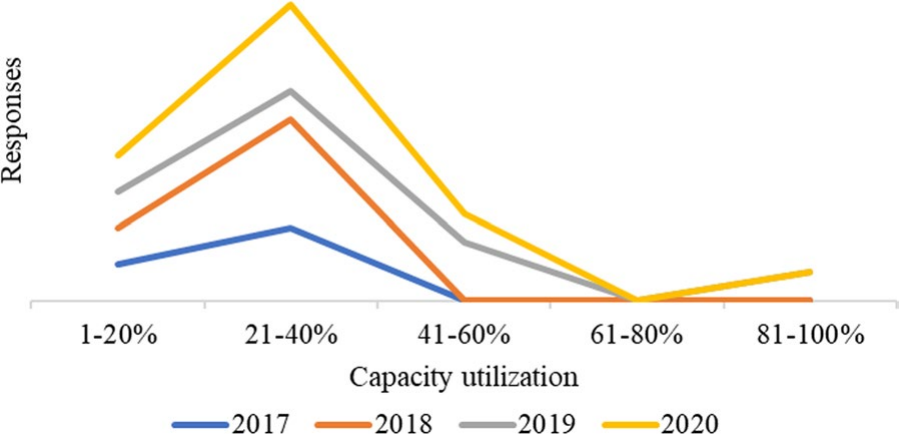
Capacity utilization of tanneries and leather goods firms in Tanzania

Capacity utilization refers to the current production level compared to what would have been produced if all the machinery/equipment were fully engaged in production activities. Based on the production capacity data acquired from the tanneries and leather goods factories from 2017 to 2020, the majority operate below their respective installed capacities with an average production capacity utilization of between 21 and 40% only (Fig. 3).

In 2017 and 2018, most leather industries (70%) utilized between 21 and 40% of their installed capacity (Fig. 3). This may have been attributed to the drop of the world wet-blue market due to the fall of China leather and leather goods exports experienced in 2016 [28]. China is among the destination countries for 90% of Tanzania's wet blue. Therefore, China's drop in imports has affected the demand for wet blue from Tanzania. In 2019, the situation didn't seem to improve much. Few industries managed to utilize between 80 and 81% of their installed capacity. These are SMEs that produce vegetable tanned leather for artisan leather products.

**Fig. 4:**
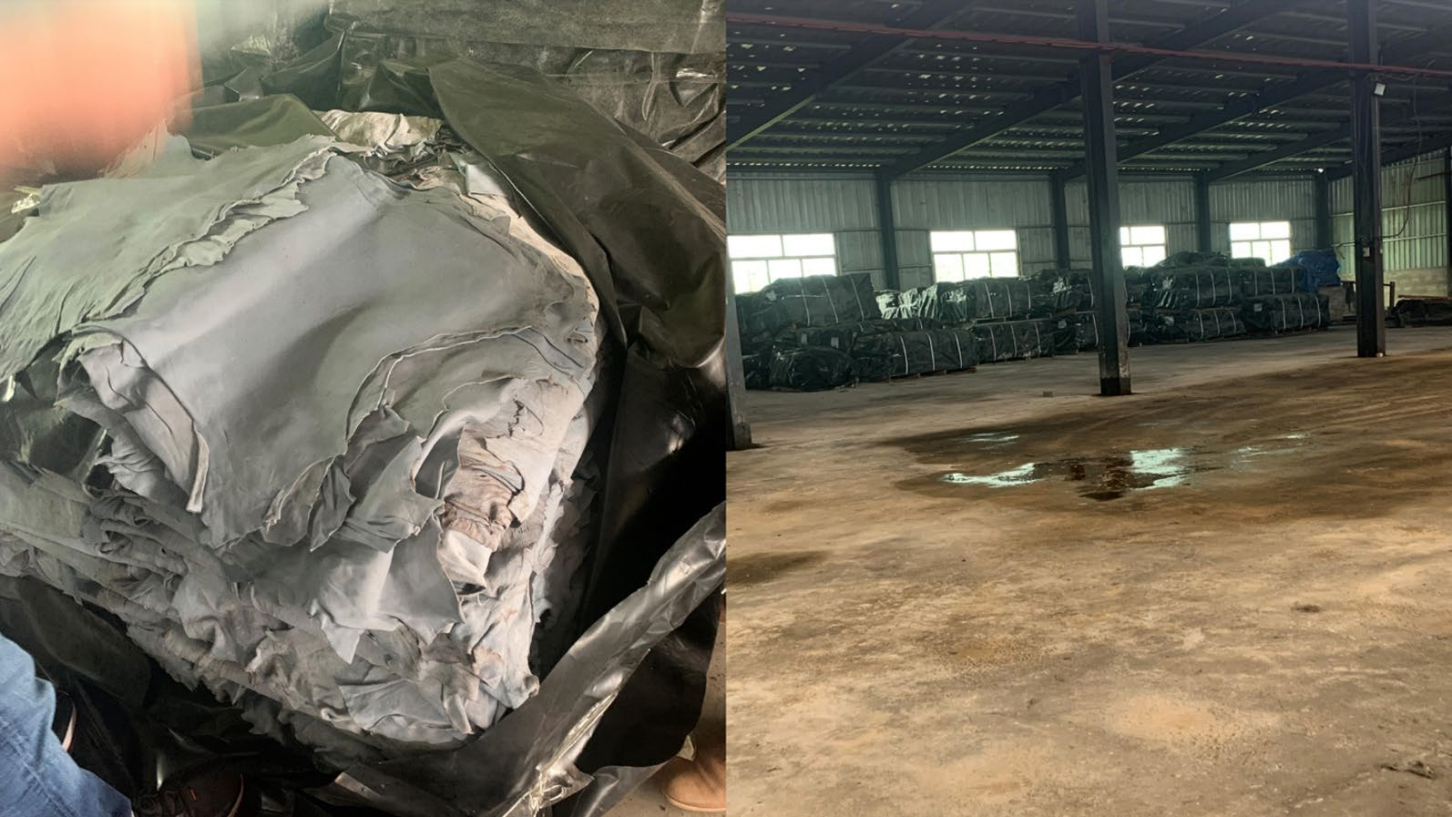
Stock of wet blue leather in one of the tanneries in Tanzania

It was noted that larger tanneries were negatively affected by the COVID-19 pandemic, which impacted the availability of imported chemicals and the market for wet blue. However, the impact is not depicted from the data in Fig. 3, which might be because during survey when tanneries reported the difficult in importing chemicals, they already had a stock purchased in bulk. They were able to continue processing using existing stock. The effect is vividly experienced now as two tanneries have temporarily stopped operating with huge stock of wet blue in warehouse due to lack of market. Figure 4 shows the large stock of wet blue leather in one of tanneries visited recently (April 2022) during normal industrial visits by TIRDO researchers. Other reasons causing underutilization of installed capacity include low quality of hides and skins supplied to the tanneries, obsolete technology, and unfair competition from synthetic leathers (plastic-based, leather-like materials) entering the local market.

#### Lack of experts

To produce high-quality leather, human skills, equipment and chemicals are needed. In leather goods and footwear production, additional attributes are required like high manufacturing skills, design know-how, availability of computer-aided design systems, branding, and marketing [10].

**Fig. 5:**
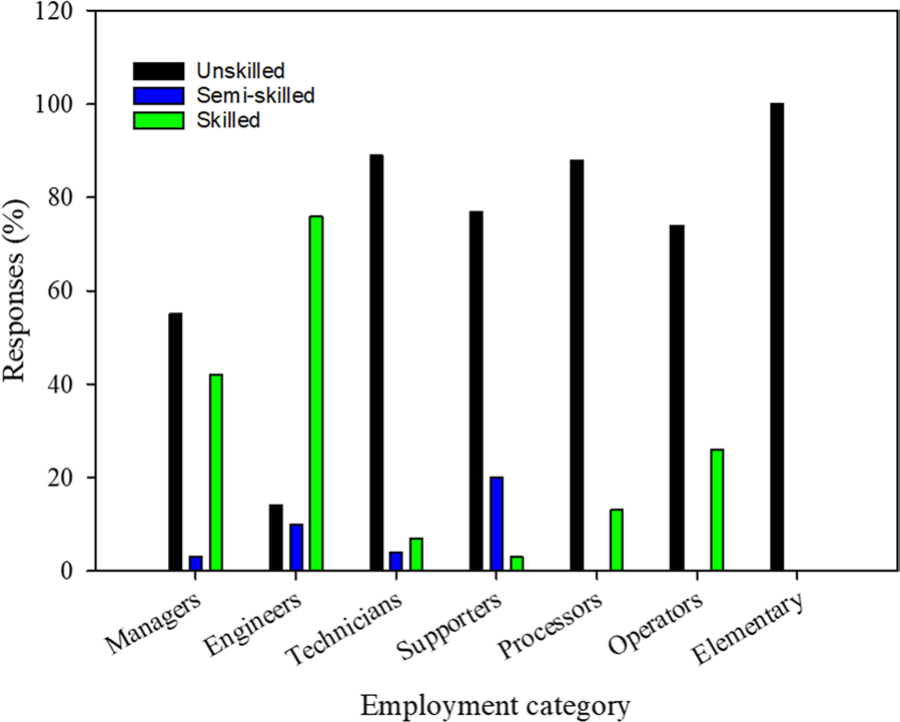
Skills level by employment category in in tanneries and leather products industries

According to Dinh et al. [29], Tanzania industrial sector mostly employs an unskilled workforce. Although an unskilled workforce is highly needed in labour-intensive industries like the leather industry, that should not jeopardize the need for skilled technical personnel to operate technical issues professionally. The workforce composition in the Tanzania leather sector in terms of skills level and employment categories revealed that more than 50% of firms' managers and 70% of the rest of employees that assume technical roles are unskilled, lacking the required qualifications to take their roles (Fig. 5). During the study and focus group discussions, tannery owners acknowledged that they face a challenge to source leather experts in the country, forcing them to hire from abroad. The scarcity of home universities that offers leather technology degrees contributes to this situation. This connotates a skill gap that needs to be the priority in the leather sector development agenda. The lack of professionals and inadequate training centres to train technicians and operators required in the sector are the causes of the prevailing situation [26, 30, 31].

As informed by stakeholders during the focus group discussion and the interview with Dar es Salaam Institute of Technology (DIT) Mwanza Campus during the field survey, some initiatives are underway. It was informed that the DIT Mwanza campus, a state-owned technical institute to develop a skilled workforce in Tanzania, is about to launch a leather processing technology and leather products technology diploma. To some extent, this initiative will alleviate the need for a skilled workforce. Lack of unskilled personnel in the Tanzania leather sector was previously reported by Andreoni [11], Kweka [12] and UNCTAD [22].

#### Poor waste management practices

**Fig. 6:**
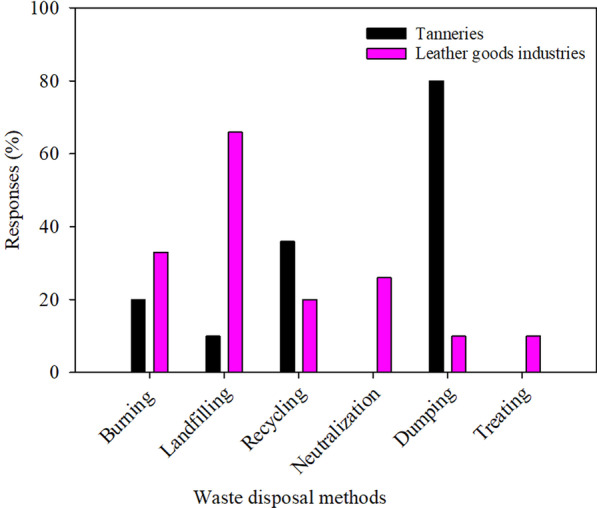
Methods used to dispose wastes

Mixed methods are used to dispose waste by tanneries and leather goods firms. Some are appropriate some are not recommended. The common methods employed are landfilling and throwing in the dumpsites (Fig. 6). Both are not recommended because they expose hazardous wastes, such as chromium-containing leather shavings and trimmings, to the environment. The recommended waste disposal method includes segregating different waste/residue fractions to facilitate recovery and re-use in manufacturing products such as pet toys and leather boards. Pre-tanning solid wastes, which include fleshings and fats, can be used to make pet food. Other means include applying sludge as compost, soil conditioner or in anaerobic digestions for energy generation. Use of sludge for manure must be done after appropriate assessment for contaminants and potential impacts to soil and groundwater. It is important to dispose non-recoverable and non-recyclable waste and sludge by proper methods, depending on the waste hazard classification [32]. According to The Environmental Management Act of 2004, hazardous wastes are classified regarding their hazardous, corrosive, carcinogenic, flammable, persistent, toxic, explosive and radioactive nature. Most of the leather waste contains chromium which is carcinogenic in nature.

#### Lack of knowledge on policies laws and regulations governing the leather sector

**Fig. 7:**
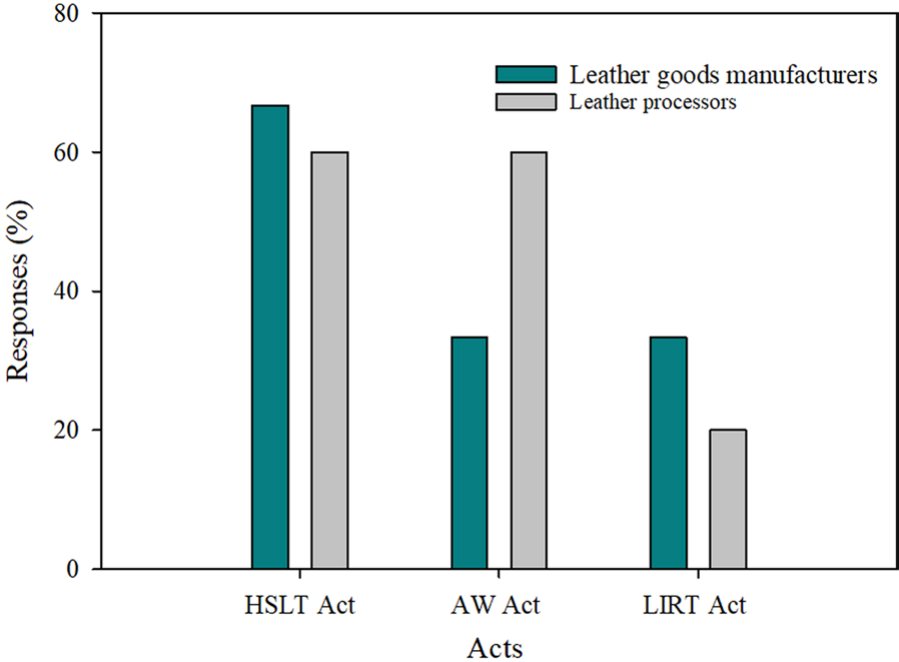
Acts regulating the leather sector in Tanzania (HSLT Act is Hides, Skins and Leather Trade Act No. 18 of 2008; AW Act is Animal Welfare Act No. 19 of 2018; LIRT Act is Livestock Identification, Registration and Traceability Act No. 12 of 2010)

The Government of Tanzania formulated several laws, regulations and policies to promote and lead the Tanzanian leather sector's growth [33]. These legislations guide the undertakings for protecting the industry and environment to make the sector sustainable. For instance, the Hides, Skins and Leather Trade Act No. 18 of 2008 clarified that any premise to be used for drying or processing hides and skins should be approved as suitable for that purpose by the inspector. However, the survey observed the existence of several small tanneries in the village processing hides and skins traditionally in the premises that are not approved for that purpose. This brings environmental pollution problems and threatens the community's health because most of them are located in the people's settlements. During focus group discussions with stakeholders, NEMC acknowledged the presence of unregistered establishments that were established without Environmental Impact Assessment (EIA). But the Environmental Management Act of 2004 requires that no industry shall be established without conducting EIA. Due to this, as informed by stakeholders during focus group discussion, some industries were established in residence areas which caused a lot of conflict with the community leading to closure of the industries due to pollution. All these are the impact of ignorance on the law, as evidenced in the findings that only 60% of leather processors and 14% of leather goods manufacturers know only Acts governing the leather sector. Nevertheless, neither leather processors nor leather goods makers among the entities surveyed know the sector's policies and regulations (Fig. 7). Unawareness about existing policies, laws and regulations governing the sector leads to non-compliance among many leather sector traders in Tanzania; hence, affecting their competitiveness, especially when trying to access the export market.

#### Unfair competition from imported plastic and second-hand shoes

**Fig. 8:**
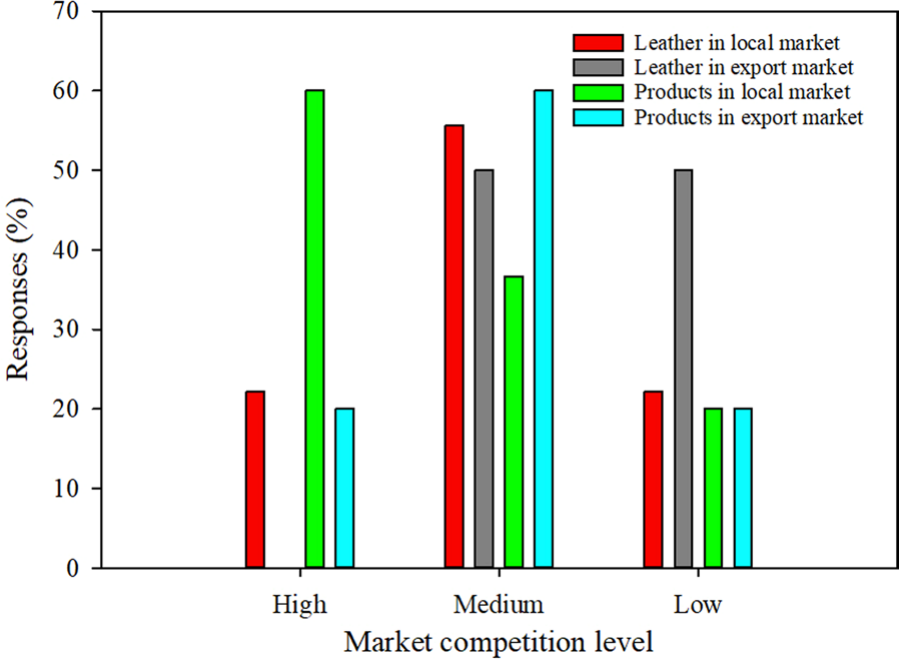
Level of competition in the local and export market for leather and leather products

Figure 8 summarizes the competition level faced by leather and leather products in the local and export markets. Leather goods face higher competition than leather in local and export markets because cheap plastic goods and second-hand products compete unfairly with local leather products. If not adequately controlled, soon the plastic products market can kill the leather products market in Tanzania and adversely affect the leather sector.

Controlling the importation of plastic and second-hand goods can trick the country's economy. This is because these businesses also contribute remarkably to the national economy and social wellbeing through employment creation. Wetengere [34] highlighted some of the benefits of second-hand products, including the increased availability of quality and fashionable and cheaper products that represented value for money, created jobs for the local people, and revenue to the Government. Apart from that, the current local footwear producers' capacity is only 1.7 million pairs per year, while the demand is about 60 million pairs per annum. Thus, wisdom is required to handle this complex form of competition to better the Tanzanian leather sector.

On the other hand, local competition for tanners is low because there are few operational tanneries in Tanzania. Among the nine large tanneries, only four are functional, while others are closed [14]. However, currently two more tanneries are temporarily closed as observed during industrial visit carried out in April 2022 by TIRDO researchers. Moreover, to some extent, tanners face competition in the export market of semi-finished leather (50%) due to low quality caused by defects on the raw hides and skins used to make such semi-finished leather.

#### Lack of awareness about restricted substances and their health and environmental effects

Various chemicals are employed in leather manufacturing to facilitate production and obtain desired properties in the finished product [35]. These chemicals include Beam-house chemicals, tanning agents, Dyeing staffs and finishing chemicals [8]. Some of these chemicals contain restricted substances such as Chlorinated phenols (PCP), Chlorinated paraffins, Chromium VI, Formaldehyde content, Dimethyl fumarate (DMF), Lead, Organostannic Compounds, Pentachlorophenol, Azo colourants and Phenylmercury Compounds, which can be retained in leather and leather products. DMF, Organostannic Compounds, Phenylmercury Compounds, Lead and PCP are considered antifungals to prevent mold growth during the transportation of leather and leather goods across countries differing in weathers [36]. Lead is regarded as an excellent colour-fastness agent [37]. Moreover, the Chlorinated paraffins are applied in fatliquoring, softening, and waterproofing, while Azo colourants are used as dyes in leather processing [8].

However, these chemical substances are persistent, bioaccumulative, and toxic to the environment. Above a certain exposure level, some Chlorophenols can be toxic to aquatic organisms and may cause long-term adverse effects in the aquatic environment. Other Chlorophenols and Organostannic Compounds have been classified as endocrine disruptors, affecting oestrogen levels and the thyroid. Pentachlorophenols, lead and chromium are known to be carcinogenic [36]. As documented by Platzek et al. [38], Azo colourants under reactive conditions cleave to produce primary aromatic amines, known as carcinogens and classified as skin sensitizers. Lead may cause irreversible neurological damage and renal disease, cardiovascular effects and reproductive toxicity [39].

Following the health threats posed by these hazardous substances, consumers are now paying more attention to the effects of those chemicals, especially their impact on human health and the ecosystem [40]. This forces tanneries to comply with a rapidly increasing set of regulations and commercial specifications, which restrict the use of chemical substances considered to have hazardous or toxic properties [16].

**Fig. 9:**
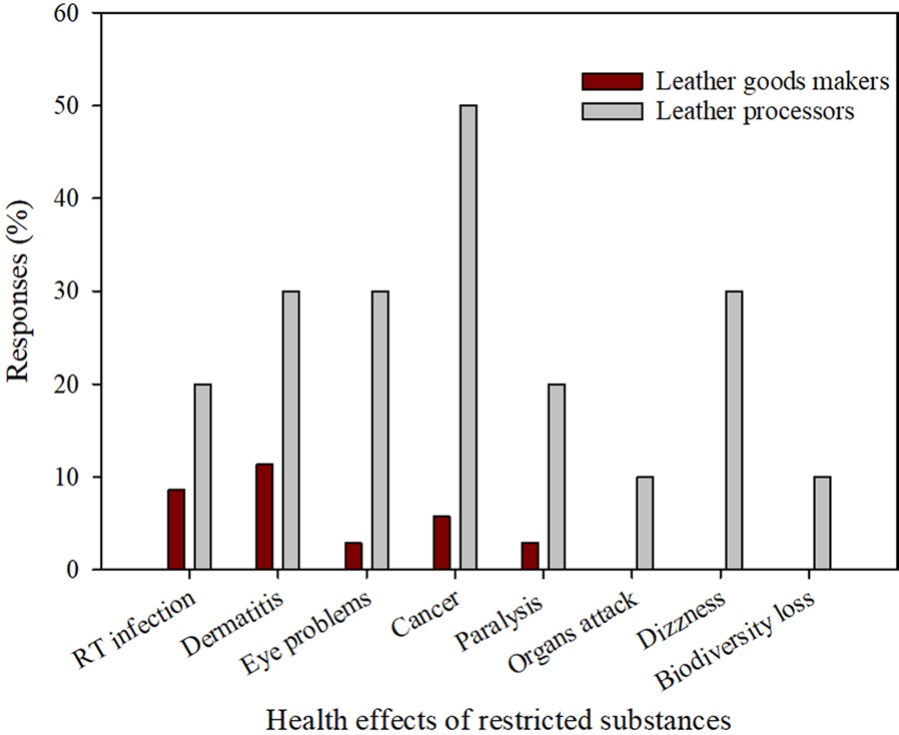
Leather and leather products manufacturers awareness about the health effects of restricted substances (RI is Respiratory Tract)

The survey shows that leather goods manufacturers in Tanzania are not aware about health effects of restricted substances in leather and leather goods compared to tanneries (Fig. 9). However, complying to restricted substances regulation is challenging even for tanneries because the existing Acts and Regulations are not very clear about the regulations of restricted substances in leather and leather goods in the country. This can be seen in the Industrial and Consumer Chemicals (management and control) Act No. 2 of 2003 and its Regulation (2004) that was enacted to control and manage the use of chemicals in the industries without harming environment and people. There is no specific regulation in the Act to guide the manufacture and use of chemicals for the leather industry, as it is in other industries like crop production. In crop production, Tanzania Pesticides Research Institute (TPRI) has formulated a regulation to guide use and management of pesticides and the list of those pesticides and their limited values are also provided. Since environmental pollution caused by hazardous chemicals used in the leather processing is of the global concern [41], Tanzania has to consider formulating specific regulations to control and manage use of leather processing chemicals as in other countries like European Union (EU), China and Japan to stay abreast with the Global leather competitive market [42].

EU countries formulated the world stringent regulations called Registration, Evaluation, Authorisation and Restriction of Chemicals (REACH) Regulation No 1907/2006 that places responsibility on all manufacturers and importers of chemicals to identify and manage the risks associated with those substances which they manufacture and market. Japan formulated regulations that restrict formaldehyde in the household products. China on the other hand formulated national standards such as GB 18,401 and GB 20,400, which limit the amount of harmful substances in consumer items. The regulations provide relevant information related to regulation and law, serve as a practical tool for compliance officers and enable companies adhere to their requirements a way to enhance their brand image.

Apart from that, awareness about harmful environmental effects of restricted substances in leather consumer goods is limited in Tanzania even at government level. This is reflected in the lack of emphasis on restricted chemicals and their hazardous effect and how to manage them in most of government documents guiding the leather sector such as strategic plans, policies etc.

The Zero Discharge of Hazardous Chemicals (ZDHC) is an organization comprised of more than 160 brands from the fashion and footwear industries to work toward zero discharge of hazardous chemicals. It was formed in 2011 to help leather industries comply with the restricted substances in leather products by ensuring safer use of chemicals throughout the supply chain [43]. The participating brands commit to comply with a Restricted Substances List and report on results from wastewater testing. In this way, ZDHC is pushing for the widespread implementation of sustainable chemistry and best practices in the textiles industry. The ZDHC Programme has identified four areas of focus to eliminate the use of hazardous chemicals [39]. These areas are Manufacturing Restricted Substances List (MRSL), wastewater quality, research and training. MRSL is a list of chemical substances that are banned from intentional use in facilities that process textile materials and trim parts in apparel and footwear [44]. The ZDHC organization encourage tanneries to download a copy of the MRSL and use this as a tool for procuring chemical formulations. Tanzanian leather and leather goods manufacturers would be encouraged to get membership to organization for increased efficiency in complying with restricted substances limits in their products.

## Conclusion, recommendations and way forward

### Conclusion

With Tanzania being among African countries endowed with plenty of livestock, the leather sector should occupy a place of prominence in its economy, given its massive potential for employment, growth and exports. Due to several reasons, Tanzania has not harnessed the full potential of its hides and skins value chain. The present study evaluated the hides and skins value chain in Tanzania to improve the performance and income from the sector. Significant constraints that hinder the sector's performance were identified, which include limited technology and innovation adoption, poor animal husbandry practices largely contribute to the low-quality hides and skins, limited capacity-building programs to the leather value chain actors and limited investments, which slows hides and skins value addition and trade on leather products.

Prioritizing solutions to these constraints by key players, policymakers and other stakeholders can bring the desired changes to the Tanzania leather sector. Notwithstanding the observed constraints, the hides and skins value chain in Tanzania possesses considerable growth potential. Therefore, Tanzania's leather sector stakeholders need to take advantage of these opportunities to revitalize the leather sector for effective and efficient performance in the local and export market.

### Recommendations

Based on the constraints and opportunities identified in the present study, the following are recommended for improvement of the Tanzanian leather sector;

i. The government needs to create a conducive and predictable business environment to attract both domestic and foreign investors in the leather industry by providing them with appropriate incentives such as tax holidays, land provision to investors at a lower lease rate, credit provision especially to local investors—hence this will lower cost of doing business and attract competitiveness. Given that Tanzania has a large number of livestock-associated with few leather industries, there is need for more modern leather industries in the country which can be obtained through encouragement of investment.
ii. The Government has to review the law, regulations, policies and quality standards to reflect the global perspectives, especially in the restricted substances compliance and to alleviate the current challenges facing the sector, such as the unfair competition from imported second-hand shoes and plastic products. The Government should also consider institutionalizing the leather sector for accountability and sustainability. The emphasis has to be in granting full mandate and support to the Leather Association of Tanzania (LAT) to carry out her duties in developing Tanzania's leather sector. The revised law, regulations, policies and quality standards must be effectively communicated to the stakeholders for effective enforcement.
iii. It is important to promote and upgrade of technology and innovation in the management and processing of hides and skins as well as in processing. This can be effectively implemented by promoting innovation through the provision of incentives in the acquisition and utilization of environmentally friendly practices and technologies including modern slaughter, flaying, processing and storage techniques. As the country is emphasizing value addition coupled with changes in technology, upgrading of technology is inevitable.
iv. The Government needs to strengthen the policy and regulatory framework to promote the employment of modern, efficient and green technologies that minimize wastes or encourage waste recycling practices such as the production of leather board, gelatin, animal feed from leather industry solid wastes.
v. Instituting and capacitating actors such as TBS in the value chain quality management framework to enhance the management of restricted substances in leather and leather goods manufactured in Tanzania and imported to Tanzania.
vi. Public and private stakeholders need to strengthen business to business linkages among leather and leather goods manufacturers to foster awareness and increased compliance to international sanitary and phytosanitary measures and standards including the EU’s REACH regulations.
vii. Special provisions are highly needed to promote the growth of leather industries. This could be done by banning export of raw hides and skins to boost value addition growth which is more likely to fetch more earnings for the citizens as well as the government. Other policy actions may include increase in export tax on semi-processed leather (wet blue) and unfinished leather (crust). This shall promote production and trade of finished leather which is advantageous in terms of securing more foreign earnings.
viii. There is a need to conduct a feasibility study to assess the possible establishment of leather chemicals industries and accessories industries in Tanzania. Investment in chemical industries that utilize locally available raw materials to produce chemicals such as fatliquors, common salts, bating agents, wetting agents, sodium bisulphite, and chromium salts would remarkably reduce operation costs and promote leather sector's competitiveness. Accordingly, investment in local manufacturing accessories such as soles, eyelets, toe puff and insoles, is equally important.
ix. Capacity building and skills development for the technical aspect of the leather is highly recommended. Training institutions such as the Vocational Education Training authority (VETA), Dar es Salaam Institute of Technology (DIT) and Sokoine University of Agriculture (SUA) need to be strengthened with the necessary expertise and infrastructures to generate skilled workforce with gender equality consideration to avoid underrepresentation of women in leather industry. These trainings need to start from the production stage including good animal husbandry practices, efficient and effective handling and collection of hides and skins, storage and preservation of hides and skins up to the final stage of the chain. The trainings will equip value chain actors with updated technical and innovative skills and knowledge on the necessary requirement needed to meet regional and global market standards and quality to stand competition.

## Data Availability

Not applicable.
